# Local avian density influences risk of mortality from window strikes

**DOI:** 10.7717/peerj.2170

**Published:** 2016-06-23

**Authors:** Ann M. Sabo, Natasha D.G. Hagemeyer, Ally S. Lahey, Eric L. Walters

**Affiliations:** Department of Biological Sciences, Old Dominion University, Norfolk, Virginia, United States

**Keywords:** Anthropogenic, Window collisions, Bird fatalities, Migratory birds, Taxonomic susceptibility, Virginia

## Abstract

Up to a billion birds die per year in North America as a result of striking windows. Both transparent and reflective glass panes are a cause for concern, misleading birds by either acting as invisible, impenetrable barriers to desired resources, or reflecting those resources over a large surface area. A high number of window strikes occur during migration, but little is known about the factors of susceptibility, or whether particular avian taxa are more vulnerable than others. We report on a study of window strikes and mist-netting data at the Virginia Zoological Park (Norfolk, Virginia, USA), conducted in the autumn of 2013 and 2014. We focused on three factors likely to contribute to an individual’s predisposition to collide with windows: (i) taxonomic classification, (ii) age, and (iii) migrant vs. resident status. Thrushes, dominated by the partial migrant American Robin (*Turdus migratorius*), were significantly less likely to strike glass than be sampled in mist nets (χ^2^ = 9.21, p = 0.002), while wood-warblers (Parulidae) were more likely to strike than expected (χ^2^ = 13.55, p < 0.001). The proportion of juveniles striking windows (45.4%) was not significantly different (χ^2^ = 0.05, p = 0.827) than the population of juvenile birds naturally occurring at the zoo (48.8%). Migrants, however, were significantly more susceptible to window strikes than residents (χ^2^ = 6.35, p = 0.012). Our results suggest that resident birds are able to learn to avoid and thus reduce their likelihood of striking windows; this intrinsic risk factor may help explain the apparent susceptibility of certain taxa to window strikes.

## Introduction

Building collisions are a major anthropogenic threat to many avian taxa, with the best estimates of mortality ranging between 365 to 988 million individuals killed annually in the United States (Loss et al., 2014). Urban structures with a high percentage of glass surface area within migrant stopover habitat typically result in high window strike mortalities during spring and autumn migration (Borden et al., 2010; Hager et al., 2013; Cusa, Jackson & Mesure, 2015; Ocampo-Peñuela et al., 2016).

While window collisions are well documented, the reasons why birds collide with structures are less understood. Even though species-specific patterns of window-strike susceptibility have long been noted, these studies have often lacked a systematic approach to surveys of local populations (Willett, 1945; Dunn, 1993; Bayne, Scobie & Rawson-Clark, 2012). Recent meta-analyses of window strike data have found strong support for increased species-specific risk to fatal window collisions when accounting for overall population abundances and species ranges, with migratory taxa being particularly vulnerable (Arnold & Zink, 2011; Loss et al., 2014). Without accounting for local densities, however, these apparent species-specific vulnerabilities may still be a result of local population density rather than true vulnerability (Kahle, Flannery & Dumbacher, 2016), although one recent study found that adult birds were less likely to hit windows despite proportionally higher abundance during the summer breeding season (Hager & Craig, 2014).

Although both migrant and resident birds are at risk of colliding with windows, the frequency with which migrant species strike windows during migratory periods is comparatively higher at buildings with a high window surface area (Dunn, 1993; O’Connell, 2001; Klem et al., 2009; Ocampo-Peñuela et al., 2016). Almost all fatal collisions that occur in urban areas involve migratory taxa, whereas window collisions in suburban or rural areas are more likely to involve resident species (Loss et al., 2014, but see Hager et al., 2013). Because migrants account for increased collision rates in urban areas during migration, it is important to explore patterns affecting susceptibility within this set of migrant populations at urban stopover sites (Klem, 1989; Cusa, Jackson & Mesure, 2015).

Many passerine species cannot distinguish glass from unhindered habitat, suggesting visual acuity as a likely predictor of window strike susceptibility (Somerlot, 2003). Additionally, mirrored or reflective windows can reflect an image of open sky, vegetation, or water sources; misleading birds into impacting glass in an attempt to reach the habitat the window is reflecting (Evans Ogden, 1996; Machtans, Wedeles & Bayne, 2013). Much research has been conducted on factors affecting bird mortality due to window collisions, particularly as it relates to anthropogenic structures. Building characteristics influence the frequency of window strikes, with perhaps the most significant characteristic being percent glass composition. Structures containing a large percentage of glass coverage (> 45%) result in high numbers of bird-window strikes (Klem et al., 2009; Borden et al., 2010; Hager et al., 2013; Kahle, Flannery & Dumbacher, 2016). Nearby habitat has also been shown to play a role in the frequency of bird strikes, where strikes increase when vegetation is located in close proximity to windows (Gelb & Delacretaz, 2009), when glass reflects an image of nearby vegetation (Klem et al., 2009; Cusa, Jackson & Mesure, 2015), or when food sources are available close to windows (Bayne, Scobie & Rawson-Clark, 2012).

Susceptibility to window strikes may also vary according to an individual’s previous experience with urban environments at large scales or local features at small scales. Migrating birds may be unfamiliar with the variety of anthropogenic structures on migration routes, which may lead to regular collisions and mortalities (Evans Ogden, 1996). Resident birds in urban or suburban areas may learn to avoid structures with glass windows within their home range, a property that has been demonstrated in hummingbirds, which have the ability to evade glass panes constructed within cages (Bent, 1940; Klem, 1989). Similarly, most fledglings develop skills that increase survivorship and reproduction with age (e.g., specialized foraging; Vanderhoff & Eason, 2008). These young birds may be similarly naïve with respect to windows, and may learn to avoid these structures. Just as resident individuals may be more knowledgeable about local hazards than migrants, older individuals that have encountered windows previously may be less likely to strike glass. Juveniles likely need to learn to avoid anthropogenic structures, something that may be accomplished with increased experience and repeated exposure to such structures.

We investigated two main hypotheses of window strike susceptibility:

*Hypothesis 1*—Taxon-specific susceptibilities to fatal window collisions are likely to occur due to intrinsic factors.
*Prediction:* Window strike proportions should not be equally distributed among families, but will vary phylogenetically.
*Hypothesis 2—* Individuals are susceptible to window strikes due to lack of experience with anthropogenic structures.
*Prediction 1:* Migrant taxa will strike windows with a proportionally greater frequency than resident birds because of the former’s inexperience with anthropogenic structures in novel areas.
*Prediction 2:* Juvenile birds will strike windows with a proportionally greater frequency than adult birds because juveniles will be less experienced with anthropogenic structures.

## Materials and Methods

### Study area

**Figure 1:**
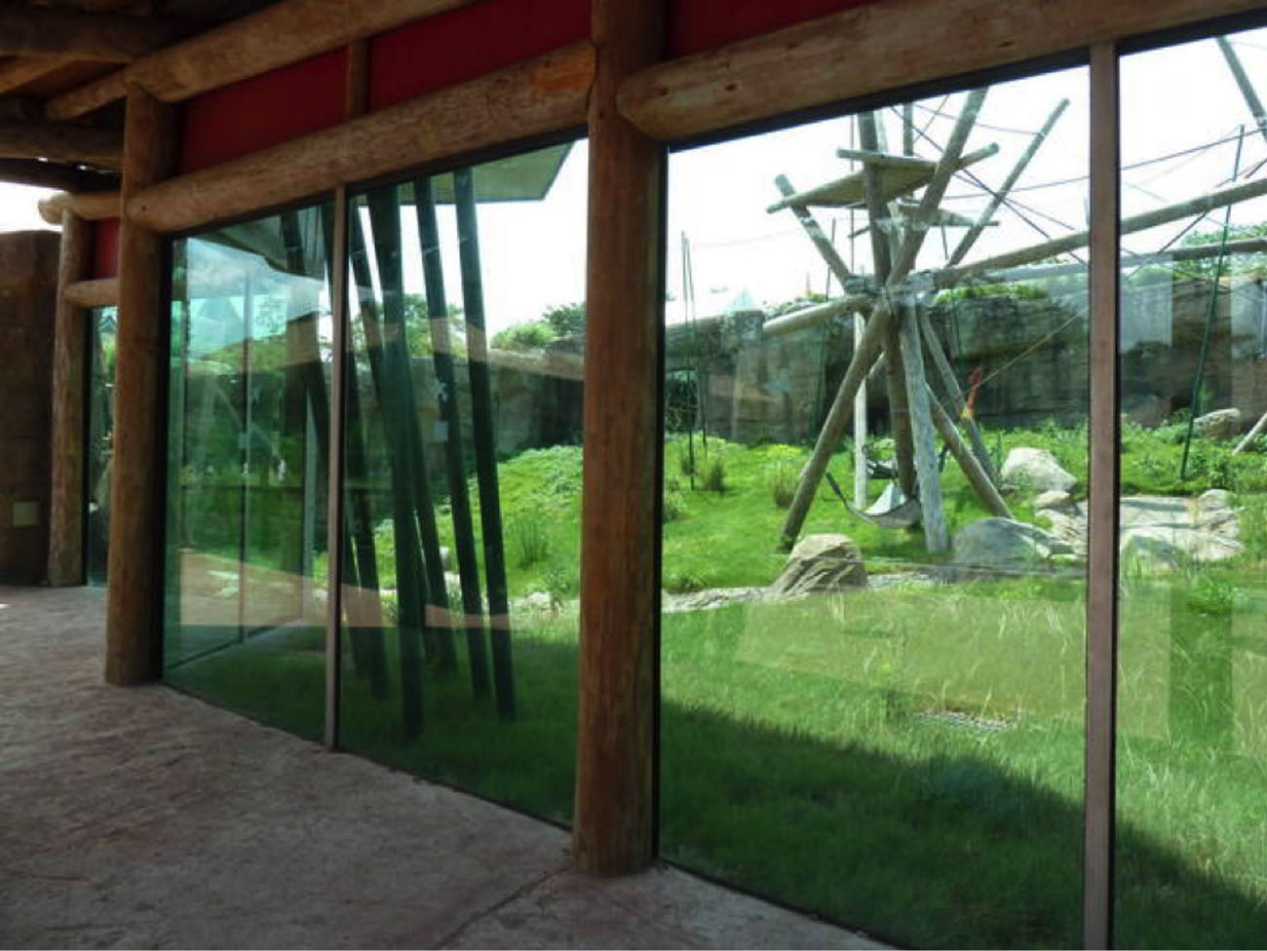
Typical view of exhibits associated with high window-strike fatalities, showing habitat beyond large, transparent glass panes.

The Virginia Zoological Park (hereafter “the zoo”) is a 53-ha urban park in Norfolk, Virginia (36°52′N, 76°16′W). Norfolk is located in coastal southeastern Virginia, bordered to the west by the Elizabeth River and to the north by the Chesapeake Bay. It is within a critically important route for migrating birds along the Atlantic Coast (Buler & Dawson, 2014). Much of the land along the Atlantic Flyway is threatened by urbanization and the erection of anthropogenic structures, resulting in the loss of potential stopover habitat for migrating birds (Valiela & Martinetto, 2007). The zoo experiences high rates of window strike mortalities during autumn migration (September–November), when these birds collide with large, transparent glass-paned exhibit windows, designed for optimum display viewing by patrons (Fig. 1).

Vegetation within the exhibits is typically open, with large expanses of grass, variable water features (including ponds and streams), and patchy distribution of shrubs and golden bamboo (*Phyllostachys aurea*). Various ornamental plants are used to obscure infrastructure, including grasses, flowering vines, wax myrtle (*Morella cerifera*), and American holly (*Ilex opaca*). Mixed deciduous trees form an open canopy in non-exhibit areas, with hedges of wax myrtle and bamboo stands used as screens between exhibits.

### Mist-netting

In order to obtain a representative sample of the proportional density of avian species using the zoo, mist-netting was conducted on a weekly basis between the hours of 07:00 and 10:00 (before the zoo was open to public interaction), from 5 October 2013 to 16 November 2013 and 21 September 2014 to 8 November 2014. This form of sampling was chosen over point counts or other visual methods to avoid biases associated with detection probability. Mist-netting is recognized to detect less vocal, smaller, and secretive species that conceal themselves in thick understory or shrubbery. It also aids in simplifying species identification, as more time can be spent to correctly identify an individual over sight and sound point counts (Rappole, Winker & Powell, 1998). Moreover, the windows surveyed for bird strikes occupied the same vertical space as mist nets (up to 3-m height), and therefore sampling bias associated with flight height would be similar for both mist nets and window mortality.

**Figure 2:**
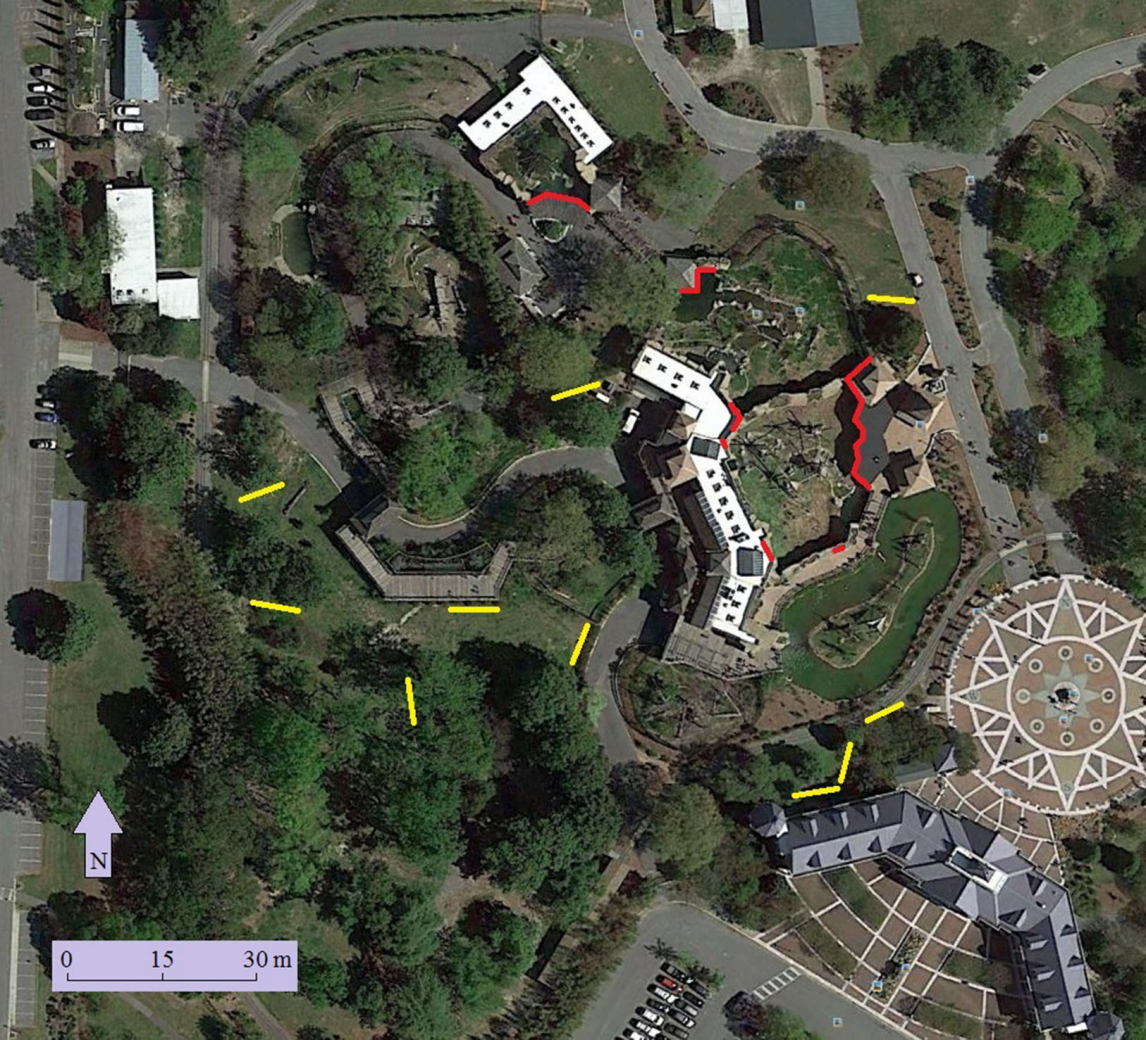
Location of windows surveyed (red) and mist nets (yellow) at the Virginia Zoo. Map data: Google, sourced from Google Earth, 36°52′40″N and 76°16′40″W, 23 April 2014.

Ten 12 × 2.5-m nylon mist nets were set up in a 1.5-ha area near the exhibit windows (Fig. 2). Mist nets were placed alongside hedges, vine-covered fences, and bamboo stands, as birds were frequently observed flying to these vegetation structures. Both 30-mm and 38-mm mesh mist nets were used to capture a range of avian taxa, from small warblers to larger mimids and jays. Nets were checked every 30 min and individuals were processed for the following information within one hour of capture: (1) identified to species, (2) age determined via plumage, skulling, and other characteristics such as eye color (after Pyle, 1997), (3) sexed when possible, (4) measured, and (5) banded. Birds were released immediately following processing.

### Window strikes

Window strike collection surveys were conducted from 5 October to 16 November 2013 and 21 September to 8 November 2014. Zoo employees conducted standardized surveys for birds that had collided with exhibit windows daily from 05:30 to 07:00. A route adjacent to windows was walked by a single observer to search for deceased birds within 3 m of each of 31 windows (1.5 × 2.8 m), inside and outside of open-air exhibits. Observers were thorough in their search, careful to check under windows and within thick vegetation for the presence of a window strike. The window under which the bird was found was recorded, and each individual was identified to species, aged, and sexed using Pyle (1997).

### Statistical analysis

Chi-squared tests were conducted to test for differences in susceptibility relative to taxonomic status (family), age, and migrant status. Migrant status was determined based on range maps in The Birds of North America Online (Rodewald, 2015); species were assigned migrant, resident, or partial migrant status based on their local presence or absence during the breeding season and/or winter. All statistical tests were conducted in R version 3.2.2 (R Core Team, 2015).

All work was carried out under the following permits: United States Department of the Interior 23803, United States Fish and Wildlife Service MB71673A-0, Virginia Department of Game and Inland Fisheries 052070, and Institutional Animal Care and Use Committee 12-006.

## Results

Over the span of 491.2 total net hours (one net hour = one hour per each mist net open), we captured a total of 314 individuals, representing ten families (Appendix A), and documented 27 fatal window strikes, representing eight families (Appendix B). All individuals mist-netted were assigned ages where possible.

### Phylogenetic status

**Figure 3:**
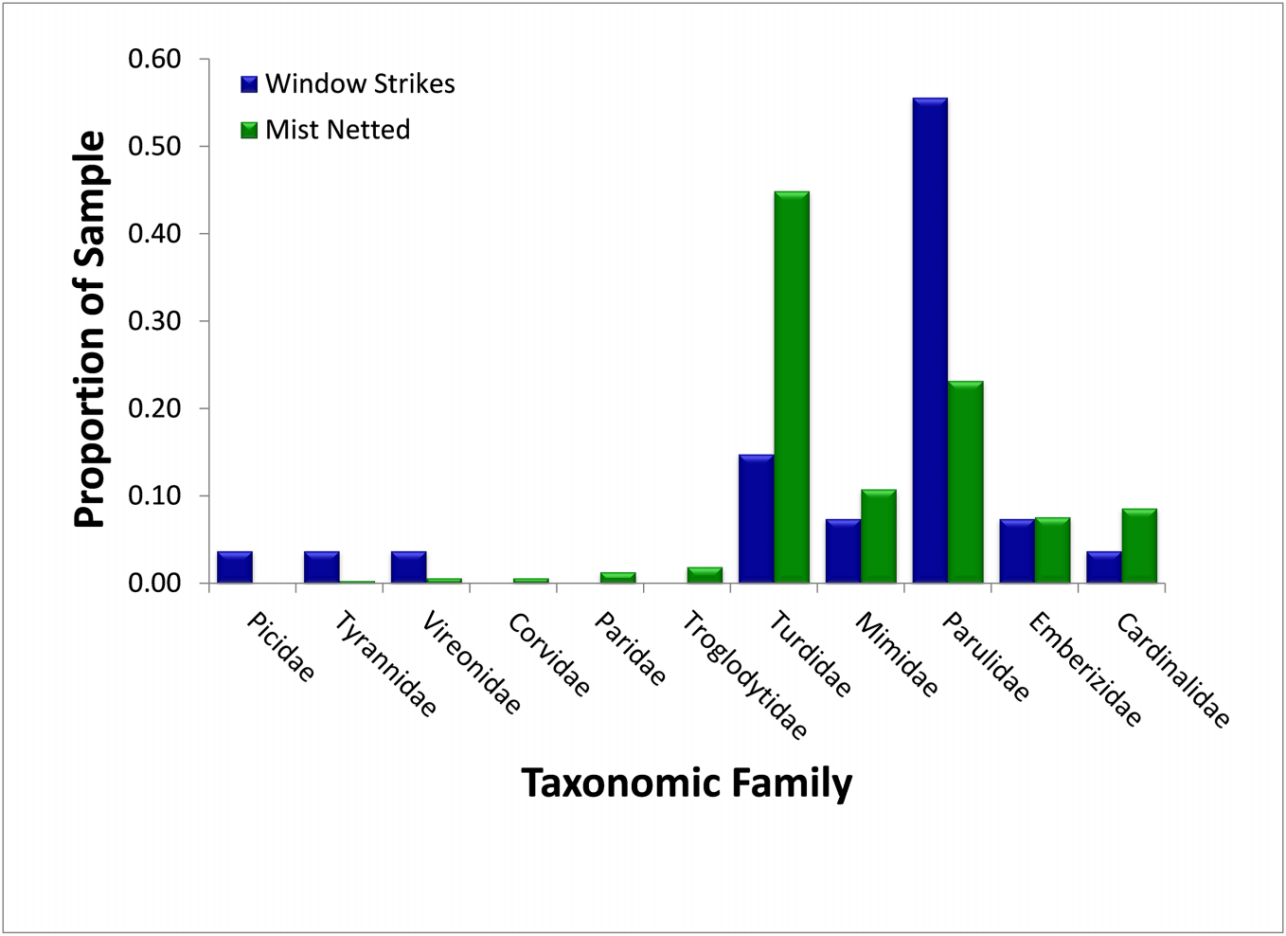
Comparison of relative abundance, as measured by mist net sampling (*N* = 314), to window strike mortality (*N* = 27), by avian family 2013–2014.

Thrushes (Turdidae) were the most numerous avian family and represented 44.9% (*N* = 314) of birds sampled using mist nets and 14.8% (*N* = 27) of fatal window strikes, and were significantly less likely to strike windows than mist-netting proportions would suggest (χ^2^ = 9.21, p = 0.002) (Fig. 3). The majority of both captured (95.7%, *N* = 141) and salvaged (75.0%, *N* = 4) thrushes were American Robins (*Turdus migratorius*). Wood-warblers were the second-most numerous avian family captured (23.3%, *N* = 314), and composed a significantly higher proportion of fatal window strikes (55.6%, *N* = 27; χ^2^ = 13.55, p < 0.001). The vast majority of wood-warblers captured (100%, *N* = 73) and salvaged (93.3%, *N* = 15) were Yellow-rumped Warblers (*Setophaga coronata*). In contrast, mimids (Mimidae), cardinals (Cardinalidae), and sparrows (Emberezidae) struck windows proportionally to their relative abundances in mist nets (mimids: 10.8% of captures and 7.4% of strikes, χ^2^ = 0.31, p = 0.578; cardinals: 8.6% of captures and 3.7% of strikes, χ^2^ = 0.79, p = 0.374; sparrows: 7.6% of captures and 7.4% of strikes, χ^2^ = 0.002, p = 0.965). The remaining six families (Corvidae, Paridae, Picidae, Troglodytidae, Tyrannidae, and Vireonidae) composed less than 5% of mist-net captures and were not used for this analysis.

### Migrant status

**Figure 4:**
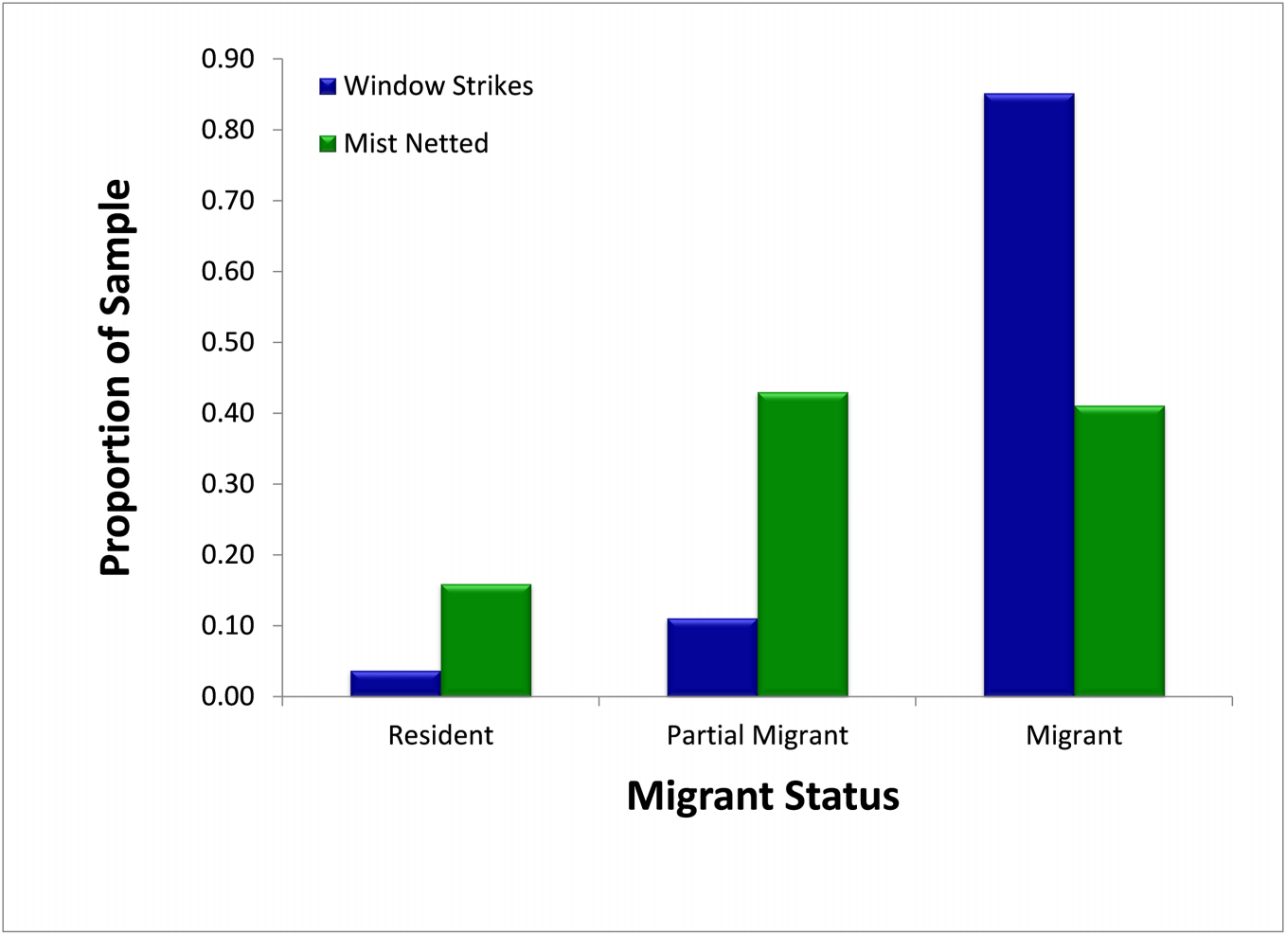
Comparison of availability, as measured by mist net sampling (*N* = 314), to window strike mortality (*N* = 27), by migratory status 2013–2014. Migrants were significantly more likely to strike windows than expected by population proportions.

A slightly higher (but non-significant; χ^2^ = 2.92, p = 0.088) proportion of migrants and partial migrants combined died from window collisions (96.3%, *N* = 27) than were sampled using mist nets (84.1%, *N* = 314) (Fig. 4). When comparing only migrants and residents, a significantly higher proportion of migrants died from fatal window collisions (95.8%, *N* = 24) than were sampled via mist nets (72.1%, *N* = 179; χ^2^ = 6.35, p = 0.012).

### Age

**Figure 5:**
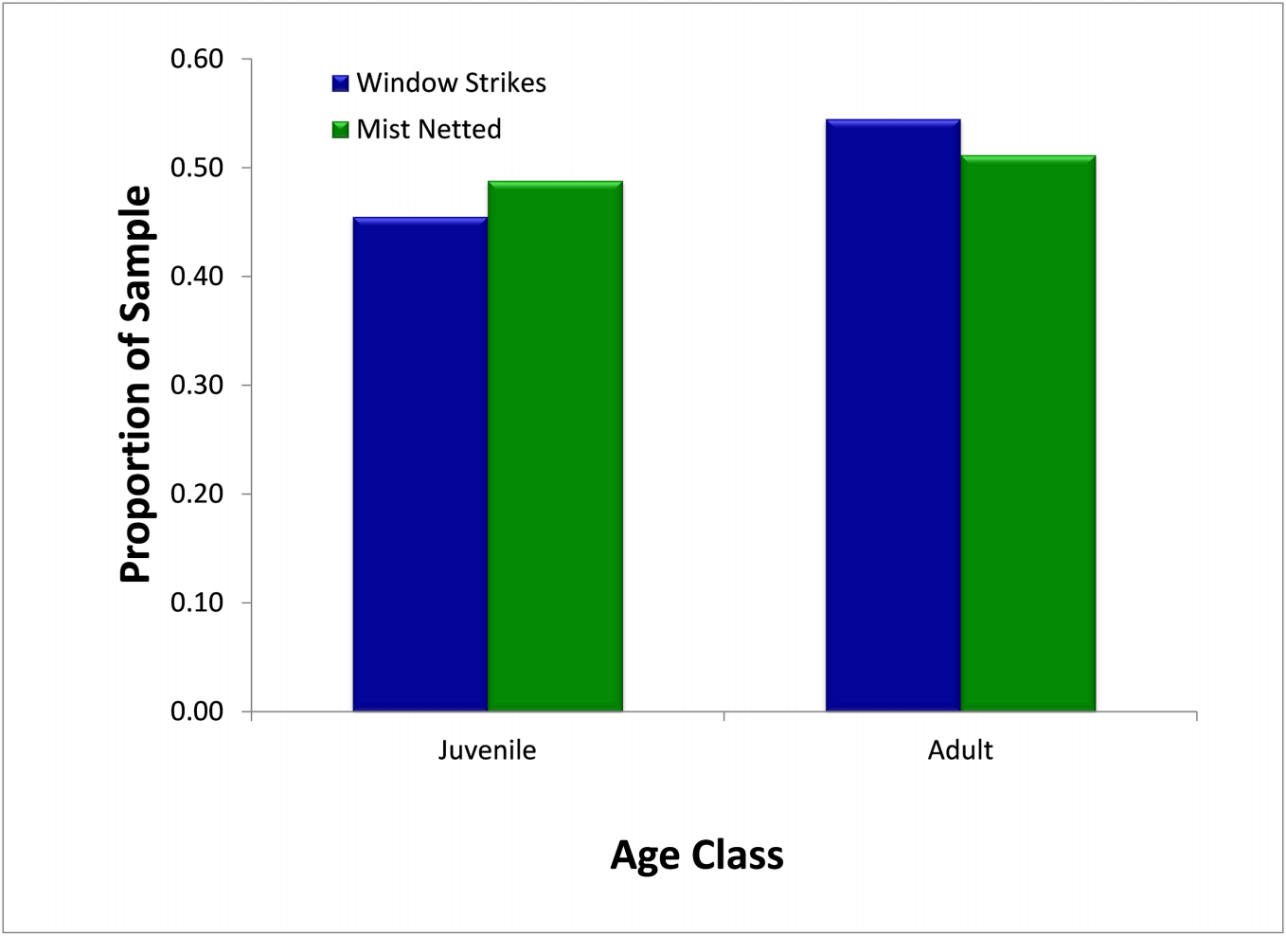
Comparison of availability, as measured by mist net sampling (*N* = 295), to window strike mortality (*N* = 11), by age, 2013–2014.

Over the study period, mist-netting resulted in the capture of a similar proportion of first-year juvenile birds (48.8%, *N* = 295) and adult birds (51.2%) (Fig. 5) Similarly, juveniles and adults comprised 45.4% (*N* = 11) and 54.6% of strikes, respectively. There was no significant difference (χ^2^ = 0.05, p = 0.827) in the proportion of first-year individuals captured in mist nets or salvaged from fatal window strikes.

## Discussion

Understanding how anthropogenic structures in urban environments affect bird mortality is an important conservation objective, as anthropogenic structures pose a documented threat to millions of individuals (Klem, 2015). As such, we examined two hypotheses regarding variability in an individual’s or a species’ susceptibility to window strikes: that susceptibility varies (i) taxonomically by family, and (ii) experientially, either by age (juveniles are either unfamiliar with local surroundings or have not experienced structures or learned of them as a danger), or by migratory status (migrants are likely unfamiliar with local surroundings and potential hazards).

**Figure 6:**
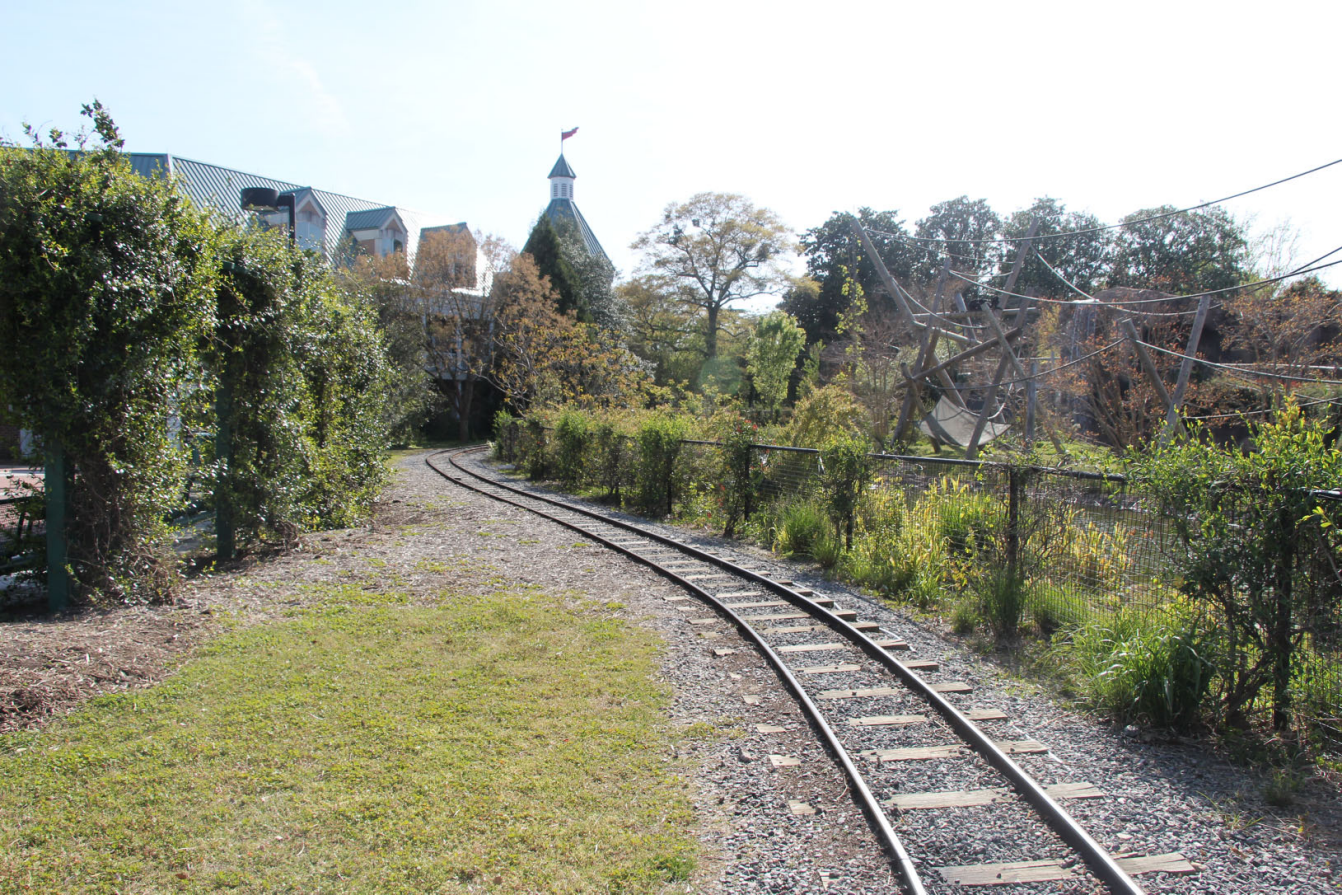
Representative habitat surrounding exhibit windows where mist-netting occurred.

In this study, Yellow-rumped Warblers were the predominant species in individuals salvaged from window strikes. These warblers are likely attracted to the Virginia Zoo as a stopover site because of the abundance of wax myrtle (Fig. 6), a common food of this species (Place & Stiles, 1992). This shrub provides a lipid-rich, nutritious fruit in the autumn and winter when other food sources (i.e., insects) are limited (Borgmann et al., 2004). Because avian density increases relative to quality of vegetation and resources (i.e., fruit, insect-rich vegetation, feeders, and water sources; Hager et al., 2013), these high quality areas generally have a higher number of window collisions (Klem, 1989). We, thus, speculate that the presence of wax myrtles near windows likely acted as an attractant for Yellow-rumped Warblers at this local site, leading to higher strike incidences in this species. Interestingly, a nearby study in North Carolina did not detect high numbers of window strikes despite Yellow-rumped Warblers being abundant during autumn and winter months (Ocampo-Peñuela et al., 2016).

Although we hypothesized that taxon-specific susceptibilities to fatal window collisions are likely to occur, our results do not fully support this claim, with the majority of families tested showing no disproportionate vulnerability to window strikes. This finding contradicts results of past studies, including several meta-analyses, which suggested that taxonomic position could predict susceptibility to window strikes (Collins & Horn, 2008; Arnold & Zink, 2011; Loss et al., 2014). The only signals for taxonomic susceptibility occurred in thrushes, which had lower susceptibility than expected, and wood-warblers, which had higher susceptibility than expected. More recent work suggests that flocking species are less vulnerable to window strikes, perhaps mediated through conspecific signaling while in flocks (Kahle, Flannery & Dumbacher, 2016), which could be a possible mechanism by which thrushes, particularly the flocking American Robins that dominated our sample, avoid window strikes. Wood-warblers, however, dominated by the flocking Yellow-rumped Warbler, were more vulnerable to fatal window strikes than expected, suggesting that flocking is not the principal mechanism determining susceptibility to striking glass.

Instead, our findings suggest that local experience of resident individuals may be an important mechanism in determining susceptibility to window strikes. While our results including the partially migratory American Robin did not show any effect of resident status, individuals in a partially migratory population likely include both year-round residents and migratory individuals, confounding the analysis. Potentially, resident individuals could be more adept at avoiding windows than migratory individuals; unfortunately, as the resident population was not marked, it was not possible to distinguish between resident and migrant status in individual American Robins salvaged from window strikes. Prior work has suggested that migratory species are particularly susceptible to strikes during their migration (Hager et al., 2008; Hager et al., 2013), potentially due to unfamiliarity with the local environment during migration. Thus, in a population with both migrants and residents, it would be expected that the resident individuals would be less likely to strike glass than migrants.

When including only migratory species in the analyses of susceptibility for resident versus migrant species, however, migrants were significantly more likely to strike windows than residents, a finding in keeping with previous studies suggesting that migrant taxa are at higher risk for fatal window collisions (Hager et al., 2008; Borden et al., 2010; Hager et al., 2013). Some studies suggest that birds are capable of using visual landmarks to navigate around previously experienced structures, often to find food sources and recover stored caches (Kamil & Cheng, 2001; Holland, 2003). When considered in the context of window collisions, this pattern suggests that resident birds are able to learn to avoid windows, reducing their likelihood of striking windows. Moreover, most strikes by resident birds are due to panic flights when close to glass (Dunn, 1993). In breeding and wintering populations without attractants such as bird feeders near windows, strikes are less common than density would predict, potentially suggesting that resident strikes are due mainly to the local density at windows and ensuing panic flights into glass, whereas migrants have notably higher risk of strikes overall (Hager et al., 2008; Kummer & Bayne, 2015).

In contrast, our results do not support the prediction that susceptibility to window strikes decreases with age. Both adult and juvenile birds were at equal risk for window strikes, giving no evidence for age-related learned avoidance of windows. The presence of fewer juvenile birds than anticipated might also be in part due to early skull pneumatization, as certain species complete skull ossification starting earlier than the time span of our study (Pyle, 1997). It is possible, then, that some birds were incorrectly classified as adults due to complete skull ossification. While we had a small sample size that may not have been sensitive enough to detect subtle age differences, similar results have been found in other studies, suggesting that age and sex are not related to the frequency of window collisions and mortalities (Klem, 1989). Studies examining breeding-season mortality from window strikes suggest that juveniles strike in proportion to abundance, while adult strikes are dominated by long-distance migrants (Hager & Craig, 2014). Our results are similar, indicating that juveniles strike in proportion to their local abundance. Other studies, however have found that a majority of strike fatalities are juvenile individuals (Hager et al., 2013; Kahle, Flannery & Dumbacher, 2016), potentially indicating a subtle age-related bias in strikes. Further work should take into account both migratory status and age to determine if these two factors interact.

While mist-netting provided a representative sample of avian species density and age distribution in our study, there is a possibility of biased mortality estimates due to scavenger-removal and survivorship. Several carcass persistence studies have shown that deceased birds are often removed at high rates by carnivores, large birds, and other scavengers (Hager, Cosentino & McKay, 2012; Parkins, Elbin & Barnes, 2015; Paula et al., 2015). The presence of carnivorous scavengers such as feral cats, for example, at the zoo could have biased the number of actual mortalities, leading to an underestimation of mortality. Another possible source of strike underestimation could be the likelihood of individuals surviving the initial collision. Not all window strikes result in an immediate fatality, with some individuals making an apparent recovery (Klem, 1990; Kummer & Bayne, 2015). If a death occurs sometime after the initial strike, there is the possibility that birds moved beyond the survey route and were thus undetected and therefore not included in total strike estimates.

In previous studies, the comparison of window strike and mist-netting abundances can result in an over- or underestimation for some species. For example *Catharus* thrushes (Gray-cheeked [*C. minimus*], Hermit [*C. guttatus*], Swainson’s, [*C. ustulatus*] and Veery [*C. fuscescens*]) were sampled in higher numbers when mist-netted, but a much lower number collided with windows. Conversely, the abundance of Northern Waterthrush (*Parkesia noveboracensis*) were overestimated in window collisions when compared with mist-netting samples (Schramm et al., 2007). While we acknowledge this possible detection bias, we found no substantial discrepancies when comparing mist-netting abundance to those of window strikes. Moreover, mist-netting is likely more representative of birds that might strike windows in this study because nets were in close proximity to windows and sampled a similar vertical space (< 3 m) to that of the exhibit windows. Other methods of sampling (e.g. area search methods) have been used to compare with window strikes (Kahle, Flannery & Dumbacher, 2016) but these methods introduce other biases and likely include species not normally prone to window strikes (e.g. Canada Goose [*Branta canadensis*] or Turkey Vulture [*Cathartes aura*]) at the Virginia Zoo. Mist-netting also allowed age to be determined more readily than any other alternative sampling method.

## Conclusions

Extrinsic risk factors such as vegetation characteristics and habitat structure likely interact with intrinsic risk factors such as experience and taxon-specific behavior to ultimately determine an individual’s propensity to strike windows. The importance of considering the influence of multiple factors is critical when considering the planning, protection, and conservation of sites that could potentially be used as stopover habitat. When resources such as food or habitat are placed in close proximity to glass structures, an increase in fatal window strikes is probable (Klem et al., 2009; Cusa, Jackson & Mesure, 2015; Kummer & Bayne, 2015). Similarly, patterns and frequencies of strikes occurring at urban locations are highly influenced by the structure and connectivity of surrounding landscapes (Longcore et al., 2012). Buildings with highly reflective windows, reflecting vegetated surroundings, are shown to have a high propensity for bird strikes (Kenney, 2015; Ocampo-Peñuela et al., 2016). Future studies should investigate extrinsic risk factors as they apply to fatal window strikes. When assessing the effects of stopover habitat quality on migratory populations, the link between arrangement and connectivity of surrounding landscape is paramount to consider, particularly as it relates to rates of fatal window collisions at stopover sites in urban environments. We encourage future research to adopt a strategy similar to ours whereby the relative abundances of birds in the surrounding environment can be compared to those striking windows, an experimental design that has been underutilized.

## Supplemental Information

10.7717/peerj.2170/supp-1 Supplemental Information 1 An appendix listing all species captured by mist nets by for fall 2013–2014.

10.7717/peerj.2170/supp-2 Supplemental Information 2 All deceased individuals collected in fall 2013–2014.

## Additional Information and Declarations

### Data Deposition

The following information was supplied regarding data availability: The raw data has been supplied as Supplemental Dataset Files.
