# Supplementary material for: Local avian density influences risk of mortality from window strikes
Source: PeerJ. 2016 Jun 23;4:e2170. doi: 10.7717/peerj.2170 (PMC4924123; doi:10.7717/peerj.2170)
Supplement: Supplemental Information 1 [file peerj-04-2170-s001.docx]

Appendix A.

| **Family** | **Common Name** | **Scientific Name** | **Migratory Status** | **2013** | **2014** |
| --- | --- | --- | --- | --- | --- |
| Cardinalidae | Indigo Bunting | *Passerina cyanea* | Migrant | 0 | 1 |
| Cardinalidae | Northern Cardinal | *Cardinalis cardinalis* | Resident | 11 | 15 |
| Corvidae | Blue Jay | *Cyanocitta cristata* | Resident | 0 | 2 |
| Emberizidae | Song Sparrow | *Melospiza melodia* | Migrant | 8 | 5 |
| Emberizidae | White-throated Sparrow | *Zonotrichia albicollis* | Migrant | 6 | 5 |
| Mimidae | Brown Thrasher | *Toxostoma rufum* | Resident | 1 | 2 |
| Mimidae | Gray Catbird | *Dumetella carolinensis* | Migrant | 8 | 13 |
| Mimidae | Northern Mockingbird | *Mimus polyglottos* | Resident | 7 | 3 |
| Paridae | Carolina Chickadee | *Poecile carolinensis* | Resident | 2 | 2 |
| Parulidae | Yellow-rumped Warbler | *Setophaga coronata* | Migrant | 42 | 31 |
| Troglodytidae | Carolina Wren | *Thryothorus ludovicianus* | Resident | 0 | 5 |
| Troglodytidae | House Wren | *Troglodytes aedon* | Migrant | 1 | 0 |
| Turdidae | American Robin | *Turdus migratorius* | Partial | 93 | 42 |
| Turdidae | Gray-cheeked Thrush | *Catharus minimus* | Migrant | 1 | 1 |
| Turdidae | Hermit Thrush | *Catharus guttatus* | Migrant | 1 | 2 |
| Turdidae | Swainson's Thrush | *Catharus ustulatus* | Migrant | 0 | 1 |
| Tyrannidae | Yellow-bellied Flycatcher | *Empidonax flaviventris* | Migrant | 0 | 1 |
| Vireonidae | Red-eyed Vireo | *Vireo olivaceus* | Migrant | 2 | 0 |
|  |  | **Total Annual Captures** |  | **183** | **131** |

An appendix listing all species captured by mist nets by for fall 2013-2014
