# Supplementary material for: Local avian density influences risk of mortality from window strikes
Source: PeerJ. 2016 Jun 23;4:e2170. doi: 10.7717/peerj.2170 (PMC4924123; doi:10.7717/peerj.2170)
Supplement: Supplemental Information 2 [file peerj-04-2170-s002.docx]

Appendix B

| **Family** | **Common Name** | **Scientific Name** | **Migratory Status** | **2013** | **2014** |
| --- | --- | --- | --- | --- | --- |
| Cardinalidae | Northern Cardinal | *Cardinalis cardinalis* | Resident | 1 | 0 |
| Emberizidae | Song Sparrow | *Melospiza melodia* | Migrant | 0 | 1 |
| Emberizidae | White-throated Sparrow | *Zonotrichia albicollis* | Migrant | 0 | 1 |
| Mimidae | Gray Catbird | *Poecile carolinensis* | Migrant | 1 | 1 |
| Parulidae | Black-throated Blue Warbler | *Setophaga caerulescens* | Migrant | 1 | 0 |
| Parulidae | Yellow-rumped Warbler | *Setophaga coronata* | Migrant | 10 | 4 |
| Picidae | Yellow-bellied Sapsucker | *Sphyrapicus varius* | Migrant | 0 | 1 |
| Turdidae | American Robin | *Turdus migratorius* | Partial | 2 | 1 |
| Turdidae | Hermit Thrush | *Catharus guttatus* | Migrant | 1 | 0 |
| Tyrannidae | Eastern Wood-Pewee | *Contopus virens* | Migrant | 1 | 0 |
| Vireonidae | Yellow-throated Vireo | *Vireo flavifrons* | Migrant | 1 | 0 |
|  |  | **Total Annual Collections** |  | **18** | **9** |

All deceased individuals collected in fall 2013-2014
